# Moonlighting with WDR5: A Cellular Multitasker

**DOI:** 10.3390/jcm7020021

**Published:** 2018-01-30

**Authors:** Alissa duPuy Guarnaccia, William Patrick Tansey

**Affiliations:** Department of Cell and Developmental Biology, Vanderbilt University School of Medicine, Nashville, TN 37232, USA; alissa.d.guarnaccia@vanderbilt.edu

**Keywords:** WDR5, WD40 repeat, epigenetics, transcription, cancer, drug discovery

## Abstract

WDR5 is a highly conserved WD40 repeat-containing protein that is essential for proper regulation of multiple cellular processes. WDR5 is best characterized as a core scaffolding component of histone methyltransferase complexes, but emerging evidence demonstrates that it does much more, ranging from expanded functions in the nucleus through to controlling the integrity of cell division. The purpose of this review is to describe the current molecular understandings of WDR5, discuss how it participates in diverse cellular processes, and highlight drug discovery efforts around WDR5 that may form the basis of new anti-cancer therapies.

## 1. Introduction

Increased understanding of the complexity of eukaryotic life has led to growing awareness of the phenomenon of ‘moonlighting’, in which a protein characterized in one context is found to play roles in other, often quite diverse, cellular processes [1]. That proteins defy neat and simple labeling is not surprising, but the mechanisms through which this occurs, and the implications it creates, are often intriguing and profound. This review is focused on WDR5, which has been extensively studied in the context of histone methylation, but more recently shown to be a preeminent cellular multitasker. The moonlighting roles of WDR5 are impressive because it is small, highly-conserved, and highly-structured, meaning that WDR5 has had to evolve functional diversity within a very constrained set of sequence and structural parameters. Here, we review current understanding of WDR5, from its canonical role in histone methylation through to functions outside the nucleus. We describe how WDR5 is able to manage its range of activities in light of extraordinary conservation, and argue that its moonlighting roles need to be carefully considered when interpreting experimental findings. We also describe how its structure, conservation, and biological connections create opportunities for small molecule-mediated inhibition of WDR5, and how its multiple roles can influence the application of these inhibitors for anti-cancer therapeutics.

## 2. Beginnings and Basics

**Figure 1:**
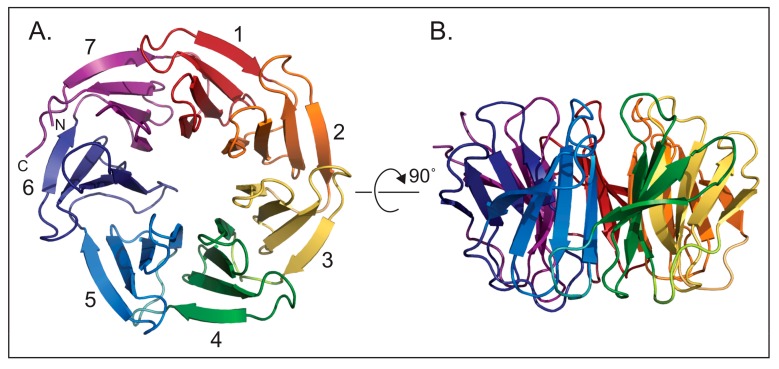
WDR5 is a seven bladed β-propeller protein. (**A**) This orientation of WDR5 displays the seven β-propeller blades of WDR5 each in a different color. The blades are numbered one to seven from the N-terminus starting with the first full blade. (**B**) Side view of the orientation of the structure in A. (PDB ID 2H14).

WDR5 first came into focus through the lens of development. Mammalian WDR5 was identified via studies of bone formation, where differential display analysis revealed induction of a ~3 kb mRNA during osteoblastic differentiation [2]. This gene was termed *BIG-3*, for BMP-2 Induced Gene 3 kb, and accurately predicted to encode a WD40 repeat-containing protein that folds into a seven-bladed β-propeller structure (Figure 1). Some years later, as the prevalence of WD40 proteins was becoming clear, *BIG-3* was renamed *WDR5* [3], better reflecting the architecture of the protein product.

Studies of skeletal development in mice showed that Wdr5 promotes cellular differentiation and proper bone formation [2,4]. Overexpression of Wdr5 in osteoblasts and odontoblasts of a developing embryo promotes growth and results in a larger than average skeletal structure [3,5], while silencing *Wdr5* in limbs of a developing embryo severely impairs bone development [6]. At about the same time that BIG-3/WDR5 was identified, its *Saccharomyces cerevisiae* homologue, Swd3, was recognized as a member of a newly-characterized histone methyltransferase complex, COMPASS (Complex of Proteins Associated with SET1), the homolog of the mammalian SET1 and MLL (mixed lineage leukemia) protein complexes that catalyze histone H3 lysine 4 (H3K4) di- and tri-methylation [7,8]. It was seminal work from the Allis laboratory, however, that connected the developmental phenotypes to epigenetics. The Allis group showed that WDR5 directly associates with methylated histone H3, the mark catalyzed by the SET1/MLL protein complexes [8,9]. They showed that depletion of WDR5 in human cells decreases expression of developmentally-essential *HOX* genes, and that whole organism depletion of WDR5 in *Xenopus* embryos causes not only a decrease in H3K4 methylation, but also severe developmental defects [9]. Further work in mouse embryonic stem cells (mESCs) cemented the connection between WDR5 and development, showing that Wdr5 expression is high in mESCs but is decreased as cells differentiate [10], and that perturbing Wdr5 expression in this context impairs differentiation and induces a sharp repression of the self-renewal transcriptional program [10]. Recognition of the importance of WDR5 to mammalian development, together with its presence in an epigenetic histone ‘writer’ complex, ushered in a new era of interrogation of the role of WDR5 in H3K4 methylation that would soon place it at the forefront of chromatin biology [11,12].

**Figure 2:**
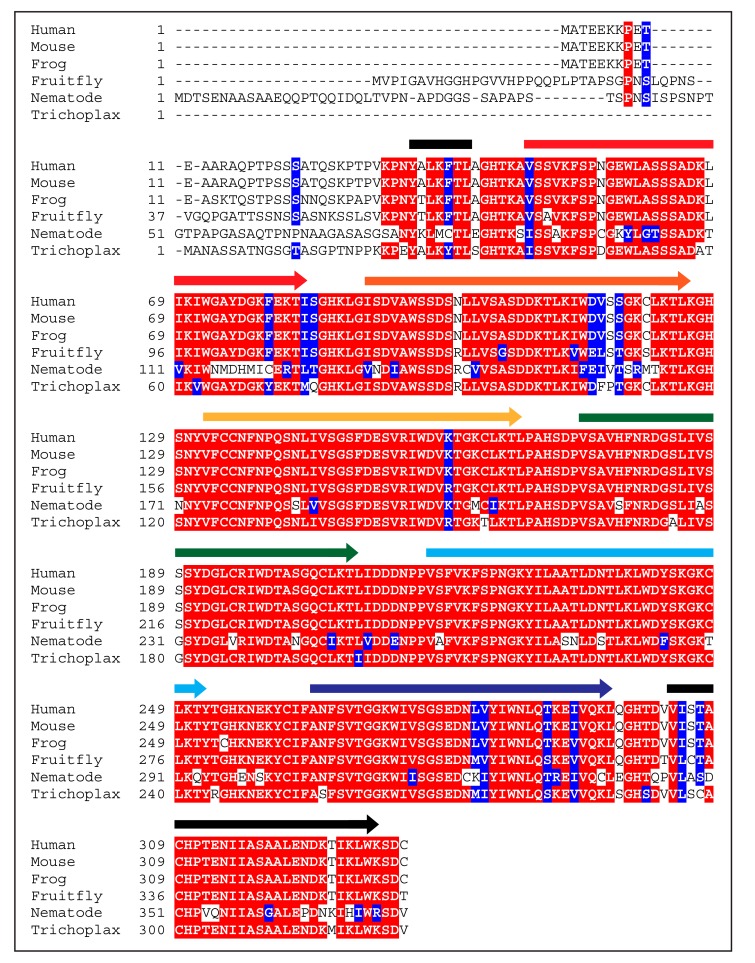
WDR5 is highly conserved in multicellular organisms. Alignment of WDR5 amino acid sequences from indicated species demonstrates high conservation of WDR5 proteins. Colored arrows above sequences indicate the residues involved in each of the seven β-propellers and match the colors in Figure 1. Residues highlighted in red are identical. Residues highlighted in blue are homologous. *Homo sapiens* (NP_438172.1), *Mus musculus* (NP_543124.1), *Xenopus tropicalis* (NP_001011411.1), *Drosophila melanogaster* (NP_524984.1), *Caenorhabditis elegans* (Q17963.1), *Trichoplax adhaerens* (XP_002109498.1).

**Figure 3:**
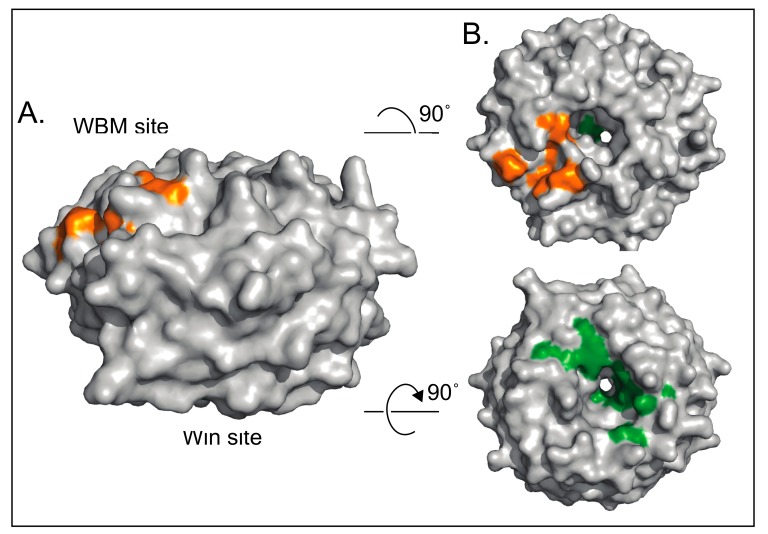
Two surfaces mediate characterized interactions with WDR5. (**A**) Surface structure of WDR5 shown from the side. In this orientation, the top face contains the “WDR5-binding motif” (WBM) site, and the bottom face contains the “WDR5-interacting” (Win) site. (**B**) Top view of the WBM site of WDR5. Residues involved in binding the WBM site are highlighting in orange: Asn225, Tyr228, Leu240, Phe266, Val268, Gln289. (**C**) Bottom view of WDR5 with residues involved in binding at the Win site highlighted in green: Ala65, Ser91, Asp107, Phe133, Tyr191, Tyr260, Phe263. (PDB 2H14).

Three basic facts about WDR5 are worth noting here. First, WDR5 is an extraordinarily highly-conserved protein. Among vertebrates, WDR5 proteins share over 90% sequence identity over their entire length [13]. Human and mouse WDR5 are identical, and venturing further afar on the evolutionary scale, basal metazoa such as *Trichoplax* have a WDR5 homologue that is ~90% identical to human WDR5 within the WD40 repeat region (Figure 2). Because of this conservation, therefore, it is safe to assume that the structure presented in Figure 1 is an accurate depiction of all extant WDR5 proteins. Second, WDR5 has been particularly amenable to structural interrogation. Over the last decade, more than 60 unique structures of WDR5 have been deposited into the Protein Data Bank (PDB), capturing WDR5 alone and in complex with co-factors and inhibitors. The extent to which WDR5 has been studied in this way reflects the importance of structural biology to understanding both the canonical and non-canonical functions of WDR5, and to developing novel inhibitors that can block interactions at the surface of the protein. And finally, although WDR5 may have more than two dozen primary direct interaction partners [14], all of the interactions that have been mapped with precision to date bind to one of two overlapping sites on WDR5 (Figure 3): a shallow cleft on one surface known as the “WDR5-binding motif” (WBM) site, and an arginine-binding cavity on the other surface referred to as the “WDR5-interacting” (Win) site. The repeating use of these two sites by various WDR5-interaction partners appears to be one of the ways that it can function discriminately in different biochemical contexts, and is a theme we shall return to as we discuss the multitude of WDR5 activities.

## 3. Function of WDR5 as a Core Member of Histone H3 Lysine 4 Methyltransferases

**Figure 4:**
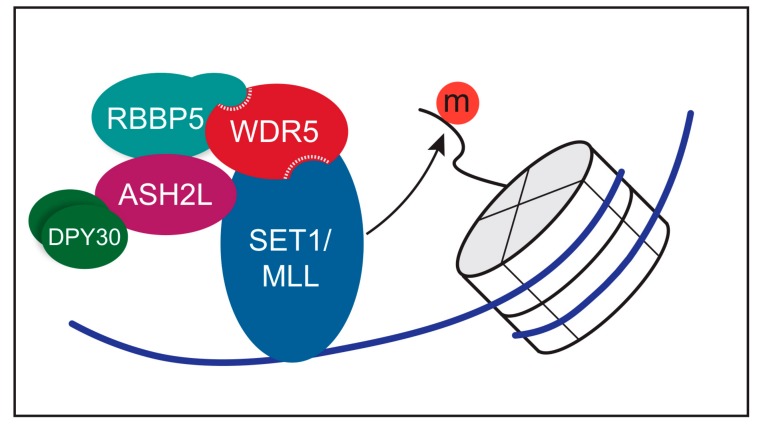
The canonical function of WDR5 is as a core component of the SET1/MLL histone methyltransferase complexes. WDR5 functions to scaffold six distinct histone methyltransferase complexes, which catalyze the epigenetic marks of mono-, di-, or tri-methylation at lysine 4 of the peptide tails of histone H3. Two binding sites on WDR5 are required for efficient scaffolding of these complexes. These six complexes differ in the identity of the SET1/MLL protein they carry.

Post-translational modifications on histones contribute to the regulation of gene expression by altering chromatin to promote active or repressive epigenetic states. Depending on the combination of marks at a particular region of the genome, different proteins are able to engage chromatin to drive processes such as transcriptional activation, transcriptional repression, and chromatin remodeling. Histone marks come in various forms including methylation, acetylation, phosphorylation, and ubiquitylation, and typically decorate the tails of histone proteins to convey an active or repressive epigenetic status. As mentioned, H3K4 di- and tri-methylation (H3K4me2 and H3K4me3) are marks of transcriptionally active chromatin, laid down by the SET1/MLL family of histone methyltransferases (HMT), also called trithorax group (TrxG), MLL-like, or COMPASS complexes [15]. The centerpiece of this review, WDR5, is a core component of these enzymes (Figure 4).

**Figure 5:**
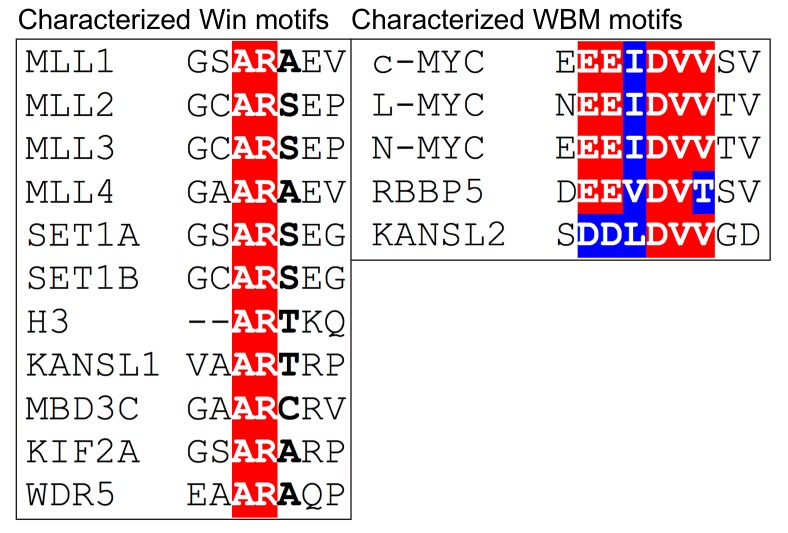
The characterized direct interacting partners of WDR5 have similar motifs. The Win motif and WBM motif sequences for WDR5-interacting proteins mentioned in this review are shown. The Win motifs are all centered on an arginine, while the WBM motifs are a specific combination of acidic and hydrophobic residues. Residues highlighted in red are identical. Residues highlighted in blue are homologous.

There are six non-redundant mammalian SET1/MLL HMT complexes, each with a distinct regulatory role [16,17,18,19,20,21] and each defined by the presence of a unique catalytic SET1/MLL subunit: SET1A, SET1B, MLL1, MLL2, MLL3, and MLL4. Besides the unique SET domain catalytic subunit, SET1/MLL HMTs are comprised of a common core set of proteins known as “WRAD”—WDR5, RBBP5, ASH2L, and DPY30 (reviewed [22])—which stimulate HMT activity above a weak basal level [23,24,25]. WDR5 plays a central scaffolding role in these complexes via its two key binding sites (Figure 3), interacting with RBBP5 via the WBM site [26], and the SET1/MLL protein via the Win site [27,28,29]. The Win site is notable here because it engages a conserved arginine within a fairly loosely-conserved Win motif [27,29,30,31,32] present in all SET1/MLL family members [27,28,29] (Figure 5). For MLL1 and SET1B, binding of the Win motif to the Win site on WDR5 is critical for robust HMT activity [33], leading to the concept that small molecule inhibition of the Win site can selectively inhibit H3K4 methylation by these two types of SET1/MLL complexes. We shall return to the issue of pharmacological inhibition of the Win site, and its likely utility in cancer, later in the review.

## 4. Moonlighting in the Nucleus

Although the actions of WDR5 in scaffolding the assembly of SET1/MLL HMT complexes are perhaps its best understood function, there is ample evidence that WDR5 functions in a multitude of processes, both in and out of the nucleus. Indeed, WDR5 associates (directly or indirectly) with nearly 200 different proteins in HeLa cell nuclear extracts and is roughly 10 times more abundant than other WRAD proteins [34], suggesting that just a small part of what WDR5 does is devoted to H3K4 methylation. Because so much of the biology of WDR5 is filtered through the lens of its HMT connection, it is important to be cognizant of its diversity of function when considering how, for example, gene knock outs or knock downs may exert their phenotypes, or how and where small molecule inhibitors may have therapeutic benefit. In this section, we will discuss moonlighting roles for WDR5 in the nucleus—and more specifically on chromatin.

### 4.1. WDR5 as a Histone Tail Reader

Studies of chromatin gradually revealed a complicated interplay between the genetic code, regulatory proteins, and post translational modifications on histones, observations that were unified by the “Histone Code” hypothesis. Put forward by Strahl and Allis, the histone code hypothesis proposes that by being “written,” “read”, and “erased” by various regulatory proteins, histone post-translational modifications enable regulation of the transcriptional state of a piece of chromatin [35,36]. Histone H3 is home to some of the most well-studied post-translational histone modifications, in particular within the first four residues of the histone tail that projects outward from the nucleosome core (Ala1–Arg2–Thr3–Lys4 or A1–R2–T3–K4). Intriguingly, as well as scaffolding the assembly of H3K4 writer complexes, WDR5 is also an H3-tail binding protein, capable of recognizing modified and unmodified H3 tail sequences via its Win site.

One of the earliest studies in this regard, by the Allis group, led to the notion that WDR5 itself is an H3K4-methylation reader [9,37]. By ‘pulldown’ assays with immobilized H3 peptides and HeLa cell nuclear extract, the authors provided compelling evidence that WDR5 in this context preferentially binds di-methylated H3K4 sequences [9]. How this occurs, however, is not yet resolved. Early structural analyses clearly show that the histone H3 tail engages the Win site of WDR5, but that Lys4 (K4) does not contact the core of the Win site of WDR5 [13,38,39,40], making direct recognition of methylation status at this site unlikely. It is possible that the mode of recognition is yet to be discovered, or that other proteins in complex with WDR5 in lysates mediate methylated lysine specificity. What this structural work did show, however, is that Arg2 (R2) of the H3 tail anchors the H3 peptide into the Win site of WDR5, where the guanidinium group of the arginine binds by π-π stacking interactions with phenylalanine residues of WDR5, Phe133, and Phe263 [38,39,40]. This “phenylalanine clamp” [38] on R2 of H3 is precisely the same mechanism through which WDR5 binds the Win motif in SET1/MLL proteins (Figure 5). The near-identical modes of Win site binding by SET1/MLL proteins and the H3 tail reveals that WDR5 cannot simultaneously bind H3 as part of an intact SET1/MLL complex [27,28,29].

**Figure 6:**
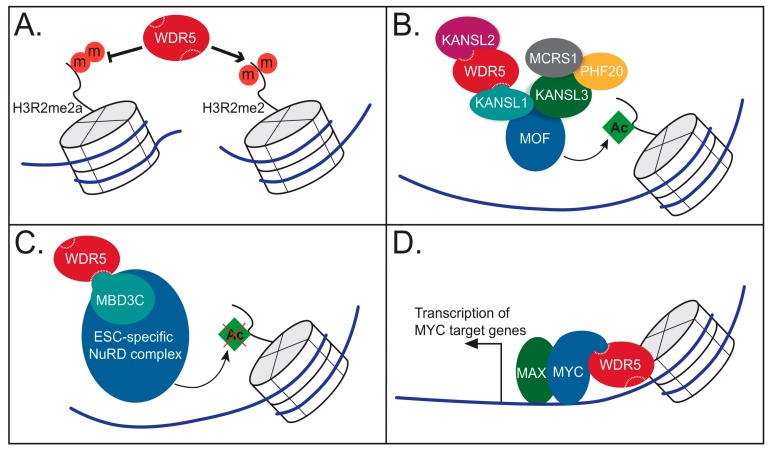
WDR5 has various roles at chromatin. (**A**) WDR5 binds directly to tails of histone H3 that are symmetrically dimethylated on Arg2. WDR5 binding is inhibited by the similar mark of asymmetrical dimethylation on Arg2. (**B**) WDR5 assembles in the non-specific lethal (NSL) complex, which acetylates histones. (**C**) WDR5 assembles in an embryonic stem cell-specific form of the nucleosome remodeling and deacetylase (NuRD) complex by binding to the MBD3C subunit. (**D**) WDR5 directly interacts with the transcription factor MYC to facilitate chromatin binding and transcriptional activation.

Although the methylation status of H3K4 may not directly influence its binding to WDR5, the methylation status of R2 in H3 does (Figure 6A). Arginine–2 of H3 is dimethylated in two distinct ways—asymmetrically, in which two methyl groups are placed on one of the terminal nitrogen atoms of the guanidino group, and symmetrically, in which one methyl group is placed on each of the terminal guanidino nitrogens. Asymmetrical dimethylation (H3R2me2a), catalyzed by PRMT6 [41], is repressive in nature, absent from active promoters and negatively correlated with the transcriptionally active H3K4me3 mark [42,43,44]. WDR5 cannot bind H3 tails that carry this repressive modification [38], which is consistent with the idea that WDR5 is generally connected to transcriptional activity. In the case of symmetrical methylation (H3R2me2), however, which is catalyzed by PRMT5 or PRMT7 [44] and marks regions of the genome that are poised for active transcription [44], binding to WDR5 is increased by more than an order of magnitude over the unmodified H3 sequence [44]. The co-crystal structure of WDR5–H3R2me2 demonstrates that these symmetrical methyl groups fill expanded space inside the Win pocket of WDR5, generating a high affinity interaction [44]. Again, as for the unmodified H3 interaction, WDR5 cannot recognize the H3R2me2 mark on chromatin within the context of an intact SET1/MLL protein complex, due to mutually exclusive binding via the Win site on WDR5.

Three important points are raised by this discussion. First, the ability of WDR5 to respond—positively and negatively—to modifications of the H3 tail strongly implies that one of its main functions in the cell is as an epigenetic reader. Second, the recurring theme of recognition of both the H3 tail and the SET1/MLL proteins via the Win site seems unlikely to be coincidental, implying an important (and yet to be discovered) functional consequence. Perhaps shared usage of the Win site allows stepwise changes in the functional state of chromatin, marking a poised gene via H3R2me2-dependent recruitment of WDR5, which can then transition at chromatin into a functional SET1/MLL complex. Or perhaps interactions at the Win site are highly dynamic, allowing WDR5 to constantly monitor the modification status of a particular segment of chromatin and respond to the marks it reads by changes in its recruitment or binding partners. Finally, the recurring re-use of the Win site by SET1/MLL proteins, the H3 tail, other WDR5 complex members (below)—and possibly by proteins yet to be discovered—issues a strong caution to interpreting the results of experimental perturbation of WDR5 (genetic or chemical) in terms of one selective function or another. Clearly, as studies of WDR5 continue, more emphasis needs to be placed on development of approaches and reagents that can tease apart the significance of the manifold interactions mediated by the Win site.

### 4.2. WDR5 as Part of the NSL Complex

MOF (males absent on the first) is a histone H4 acetyltransferase that assembles into two distinct functional complexes, the MSL (male-specific lethal) complex and the non-specific lethal (NSL) complex [45]. WDR5 associates with MOF only within the NSL complex [46,47,48,49], which also includes five other subunits: KANSL1, KANSL2, KANSL3, PHF20, and MCRS1 [47,48,49] (Figure 6B). A recent structural interrogation of NSL from the Akhtar laboratory revealed direct interactions with WDR5 that are parallel and mutually exclusive with direct interactions in the SET1/MLL complexes [50]. WDR5 directly interacts with KANSL2 and with KANSL1 at the same exact binding sites that bind RBBP5 (WBM site) and SET1/MLL enzymes (Win site), respectively [50]. The region of KANSL2 that binds WDR5 is highly conserved and contains a WBM motif that is parallel to the WBM of RBBP5 [50] (Figure 5). Similarly, KANSL1 binds to WDR5 through a highly conserved arginine-containing Win motif, analogous to the Win motifs within H3 and the SET1/MLL proteins [50] (Figure 5). At least in *Drosophila*, this KANSL1-WDR5 interaction appears to be required for efficient recruitment of the NSL complex to chromatin, as mutations that disrupt this interaction reduce NSL chromatin binding [50]. Because WDR5 is a mutually exclusive component of a histone methyltransferase complex and a histone acetyltransferase complex, it is not surprising that affinity purified FLAG-WDR5 samples contain both methyltransferase and acetyltransferase activities [51,52]. But what is remarkable is that the NSL complex functions to boost SET1/MLL methyltransferase activity, pointing to a crosstalk between these two varieties of histone modifying complexes [52]. As WDR5 is the only known shared component of both SET1/MLL and NSL complexes, it is tempting to speculate that its moonlighting here is no coincidence, and that WDR5 plays a role in coordinating the establishment of these two transcriptionally-positive histone marks. But again, the reuse of these two sites on WDR5, and the mutual exclusivity that mandates, makes it challenging to imagine a simple underlying mechanism.

### 4.3. WDR5 as Part of the NuRD Complex

Another intriguing appearance by WDR5 is within the nucleosome remodeling and deacetylase (NuRD) complex, a chromatin-associated protein complex that performs the dual-roles of chromatin remodeling and histone deacetylation. The NuRD complex originally surfaced within the context of transcriptional repression, but it is now clear that it functions in a multitude of chromatin-centric events, including gene activation and DNA repair [53]. Several of the six core proteins of the NuRD complex have paralogs or isoforms that assemble into NuRD in mutually exclusive ways, generating a number of different NuRD complexes that have cell type- or context-specific activities [53]. Wdr5 was identified as part of the NuRD complex through a proteomic analysis in mouse embryonic stem cells and confirmed to interact by co-immunoprecipitation [54]. Further interrogation of the NuRD–WDR5 connection showed that human WDR5 is brought into NuRD through the subunit MBD3 [55] (Figure 6C), which has three isoforms, only one of which—MBD3C—interacts with WDR5. It will perhaps not surprise the reader to learn that MBD3C engages WDR5 via the Win site [55], which binds an arginine-containing Win motif found only in the MBDC3 isoform (Figure 5). Interestingly, MBD3C is expressed primarily in embryonic stem cells (ESCs) and has been shown to form an ESC-specific NuRD complex that disappears as cells differentiate [55], implying that the function of this complex is to contribute to maintenance of the stem-like state.

### 4.4. WDR5 Works with Sequence Specific Transcriptional Regulators

Given that WDR5 assembles into so many different multi-protein complexes, it is somewhat surprising to learn that WDR5 can be directly contacted by sequence-specific transcription factors. Yet several such direct interactions have been described—with Oct4 [10], Twist1 [56,57], HSF2 [58], and c-MYC [59] (Figure 6D)—the majority of which have been implicated in promoting the activity of the specific transcription factor. The interaction with c-MYC, first reported by our laboratory [59], provides an illustrative example of yet another molecular function for WDR5. MYC binds directly to WDR5 via an invariant sequence motif present in all extant MYC proteins. This motif is almost identical to the WBM of RBBP5 and KANSL2 (Figure 5), and indeed structural analyses showed that MYC interacts with WDR5 via the WBM site (Figure 3) and in a way virtually identical to RBBP5 and KANSL2 [59]. WDR5 and MYC co-localize on chromatin, and mutations in MYC that disrupt the interaction do not impact the binding of WDR5 to chromatin, but they do prevent MYC from stably associating with its target genes and from driving tumorigenesis in mice [59]. Thus, the MYC–WDR5 interaction—which is conserved in the L-MYC [59] and N-MYC [60] family members—facilitates the recruitment of MYC to target gene chromatin; a non-enzymatic activity and one quite different from other proposed functions of WDR5. Additionally, MYC’s requirement for direct contact with the WBM of WDR5 to bind a multitude of sites across the genome reveals that, at any moment in time, a significant fraction of chromatin-bound WDR5 is not in its SET1/MLL- or NSL-complexed states.

### 4.5. The Ever-Expanding Nuclear WDR5 Interactome

Besides the well-described moonlighting activities mentioned above, there is a growing body of evidence, less mechanistically developed, suggesting that these functions may just be the tip of the WDR5 iceberg. For example, WDR5 associates with chromatin remodelers through its interactions with CHD8 [61,62] and INO80 [48,63]. It is part of the ATAC (Ada2a-containing) complex, which is an H4-specific histone acetyltransferase [64,65]. It is a member of a repressive histone methyltransferase complex, PRC1.6 (also called E2F6.com) ([66,67,68] reviewed [69]), and forms part of the recently described WHHERE complex (complex containing Wdr5, Hdac1, Hdac2, and Rere) [70] that functions as a retinoic acid receptor cofactor in control of embryonic symmetry. And in breast cancer cells, WDR5 interacts with the canonical PRC1 protein CBX8 to maintain oncogenic NOTCH signaling [71]. Moreover, WDR5 does not just complex with proteins, but is also able to selectively interact with long non-coding RNAs (lncRNAs), such as HOTTIP, at a site overlapping with the WBM site, to recruit WDR5 to *HOXA* genes [72,73]. WDR5 also binds the NeST [74], GClnc1 [75], and HOXD-AS1 [76] lncRNAs, although how it recognizes these RNA species has not been determined. Clearly, the multitude of protein-protein and protein-RNA interactions centered around WDR5 is large and, although a lot of work needs to be done to tease apart this litany of interactions, we can reasonably conclude that WDR5 is a highly-networked chromatin-associated protein that will continue to fascinate researchers for years ahead.

## 5. WDR5 Moonlights off Chromatin

**Figure 7:**
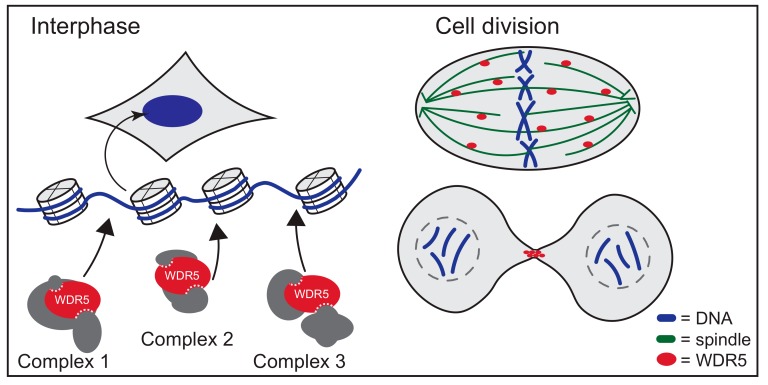
WDR5 performs roles at chromatin and roles in cell division. In addition to assembling in multiple complexes at chromatin during interphase, WDR5 functions at the spindle and midbody of dividing cells to facilitate the integrity of mitosis and cytokinesis.

Early on in the study of chromatin-modifying complexes, a curious parallel was noticed between the histone monomers of chromatin and tubulin monomers of microtubules: both assemble into polymers that are post-translationally modified in specific ways. In the case of tubulin, this phenomenon is particularly important during cytokinesis, where post-translational modifications of microtubules play a key role in orchestrating events at the mitotic spindle and the midbody [77]. Strahl and Allis predicted that, similarly to the regulation of histones, microtubules might also be regulated by post-translational modifications [35], and an analogous “tubulin code” was soon proposed [78]. What has proved quite remarkable about this analogy is that not only is regulatory information laid down in specific patterns by modifications on tubulin, but that the writers, readers, and erasers of the histone code have been found to moonlight as part of the tubulin code. There is thus a growing awareness of how epigenetic regulatory proteins are also involved in chromatin-independent roles in cell division [79], and WDR5 is no exception (Figure 7).

WDR5 localizes to the mitotic spindle and to the midbody in dividing human cells [80,81]. This localization depends on the integrity of the Win site, as mutations in this site of WDR5 prevent its stable association with the midbody [80]. Every indication is that binding of WDR5 to the midbody and spindle is functionally relevant, as these WDR5 Win site mutants also fail to rescue mitotic defects associated with WDR5 knock-down [82]. Proteomic screening for cytoplasmic partners of WDR5 led to the identification of KIF2A, a kinesin motor protein, as a direct WDR5 interaction partner [81]. KIF2A carries a Win motif that appears to directly bind the Win site on WDR5 [81] (Figure 5). Interestingly, both the Win motif of KIF2A and the Win site of WDR5 are required for KIF2A recruitment to the midbody, suggesting that KIF2A and WDR5 work together to recruit each other to this location. Again, the sheer volume of traffic at the Win site of WDR5 makes interpreting results of these kinds of mutational experiments challenging, but the Win site-dependent mitotic localizations of WDR5 clearly point to a functional role for WDR5 in regulating cell division. This notion is further reinforced by a few studies that—while not looking explicitly at WDR5—have shown that WDR5-containing epigenetic modifier complexes such as NSL [83] and ATAC [84] associate with microtubules and play key roles in mitotic integrity. Mechanistic understanding of how WDR5 functions in these roles is clearly needed, but it appears safe to say that WDR5 has important functions on and off chromatin, and all of these need to be considered and understood to fully appreciate the breadth of its cellular activities.

## 6. Networking with WDR5

On the surface, WDR5 is nothing special. Its architecture—WD40 repeats forming seven β-propellers—is shared by many scaffolding proteins of multisubunit complexes [85]. Indeed, the WD40 repeat domain is one of the top ten most common interacting domains across eukaryotic proteomes [86]. With so many WD40 proteins to go around, we might expect that each chromatin regulatory complex, for example, might have a dedicated WD40 family member. Yet we see WDR5 being used in multiple complexes and in multiple ways, a fact that raises two intriguing questions: Why is this one protein repurposed so many times and how can it do so many things without creating cellular confusion?

It is possible that the moonlighting of WDR5 in multiple complexes is a mechanism that cells use to coordinate broad changes in the activity of different epigenetic regulatory complexes. This coordination, for example, could allow multiple WDR5-containing complexes to be regulated simultaneously, such as in ESC differentiation where down-regulation of WDR5 [10] would be expected to alter the function of both the MLL1/SET and NuRD complexes that have helped keep ESCs in their stem cell state. Or the coordination could be more local, allowing a single segment of WDR5-bound chromatin to transition between different histone modification states, with WDR5 switching out its entourage of interaction partners to drive different patterns of histone methylation or acetylation. Or it is possible that the repurposing of WDR5 is a consequence of evolution, of it being part of some primordial chromatin modifying complex that evolved and specialized around a common WDR5 core.

As to how WDR5 manages its diverse extracurricular activities, a clue may come from the repeated reuse of the WBM and Win sites. It is intriguing to note that for all direct WDR5 interaction partners that have been studied in detail the interactions occur through either the Win or the WBM sites. Despite extraordinary conservation of residues in WDR5 (Figure 2)—conservation that far exceeds what one might reasonably expect to be required to maintain the structural integrity of the protein—only these two sites have ever been shown to mediate interaction of WDR5 with another molecule. It is probable that proteins capable of binding the “sides” of WDR5 are yet to be discovered, or that interactions with known proteins also include residues beyond the WBM or Win sites. But it is also intriguing to consider that reuse of these sites may play an important bookkeeping role. WDR5 that has bound RBBP5 or MLL1, for example, could be neatly marked for assembly into a SET1/MLL-type complex, with little chance that it could errantly associate with NSL proteins or members of the ATAC complex, due to the overlapping binding surfaces involved. In a somewhat counter-intuitive way, therefore, the rather limited way that WDR5 interacts with its many different partners may be the secret to its successful moonlighting activities.

## 7. WDR5 and Drug Discovery

In the last decade, it has become apparent that epigenetic regulatory proteins can be targeted by small molecule inhibitors for therapeutic benefit. There are currently dozens of small molecule epigenetic inhibitors in various stages of clinical trial in the United States [87], targeting histone code writers, readers, and erasers, and it is likely that this number will continue to blossom in the years ahead. For WDR5, inhibitor discovery efforts are still in their early phases, and it is no surprise that discussion in this area centers on the two sites that have come up continually throughout this review. For example, we have proposed that WBM site inhibitors may have utility as anti-cancer agents, by virtue of their ability to block the MYC–WDR5 interaction, thwarting MYC function in cancer cells [59]. This concept was further expanded on by Draetta and colleagues [88], who showed that the MYC–WDR5 interaction is a critical determinant of pancreatic cancer, and provided a strong rationale for how WBM inhibitors could synergize with ATR inhibitors for therapeutic effect in this context. As yet, no small molecule WBM site inhibitors have been reported, but it seems that it is just a matter of time before these become available.

For the Win site, however, two types of inhibitors have been discovered. One Win site inhibitor, MM-401 [89], is a macrocyclic peptidomimetic, which (as the description implies) engages the Win site of WDR5 by mimicking the arginine of the Win motif. MM-401 binds the Win site with relative high affinity (*K_d_* ~ 1 nM) and inhibits the HMT function of MLL1 complexes in vitro, consistent with the requirement of the MLL1–WDR5 interaction for robust methyltransferase activity [33]. Dou and co-workers originally proposed that Win-site inhibitors would have efficacy against tumors bearing rearrangements of the *MLL1* gene (MLLr), a common occurrence in acute myelogenous leukemia (AML). This concept was, in turn, based on the idea that MLLr cancers almost always retain one wild-type copy of *MLL1*, and are uniquely dependent on the HMT activity of wild-type MLL1-complexes for survival [90]. And indeed MM-401 appears to be highly selective against MLLr cancer cells and cell lines in vitro [89], where it depletes H3K4 trimethylation at *HOXA* genes, driving cellular differentiation and apoptosis. Subsequently, however, it became clear that MLLr cancers are not dependent on MLL1, but instead rely on MLL2 [91]—notable in this case because, although MLL2 complexes with WDR5, it does not depend on Win site binding for methyltransferase activity [33]. It seems unlikely, therefore, that the mechanism of action in this instance is due to inhibition of MLL1-mediated H3K4 methyltransferase function, raising the question of how MM-401 selectively inhibits MLLr cells.

The Structural Genomics Consortium has generated a set of more traditional small molecule inhibitors of the Win site [92,93,94,95,96,97], one of which, OICR-9429 (*K_d_* ~ 100 nM), inhibits MLL1-HMT activity in vitro and shows inhibition of cancer cell lines in culture. Interestingly, the impact of OICR-9429 on MLLr cancer cells has not been reported. But it has been shown to inhibit AML cells that carry the p30 isoform of the transcription factor C/EBPα [94]. The rationale for inhibition in this context is that the oncogenic p30 isoform of C/EBPα binds better to WDR5 and WDR5-containing complexes than non-oncogenic isoforms, but how p30 C/EBPα binds WDR5, whether this is through a Win site engagement, and how this connects to HMT activity are all unknown. A second use for OICR-9429 was demonstrated in seminal work from the Berger laboratory [98], who showed that cells expressing gain-of-function (oncogenic) p53 mutants—which comprise the largest group of *TP53* mutations in human cancer—are uniquely sensitive to OICR-9429. Here, there is no direct contact between oncogenic p53 variants and WDR5, but rather sensitivity to compound appears to result from selective induction of MLL1 expression by the oncogenic p53 variants, the impact of which is mitigated by Win site blockade and inhibition of MLL1-HMT activity. Given the magnitude of involvement of p53 gain of function mutants in cancer, these findings open the door to a potentially huge therapeutic impact of a drug-like Win site inhibitor.

The examples of MM-401 and OICR-9429 raise two important issues. First, it is not always clear why or how a Win site inhibitor will block cancer cells. The premise on which MM-401 was established has been challenged, and yet it clearly results in selective inhibition of MLLr cells in culture. We do not know how WDR5 makes contact with p30 C/EBPα, yet targeting the Win site of WDR5 can inhibit cells bearing this oncogenic C/EBPα variant. And there is no direct contact between p53 gain-of-function mutants and WDR5, yet OICR-9429 appears highly effective in that setting. These examples suggest that, despite all that is known about WDR5, the application of Win site inhibitors may best be determined empirically. Perhaps this is not surprising, given the extreme multi-tasking that WDR5 can perform. Potent Win site inhibitors would likely displace all proteins that engage this site on WDR5, and it may very well be that the particular Win binding protein that predominates in any given context, or other perturbations in a cell that render it sensitive to loss of a particular Win interactor, determine whether a cell lives or dies in response to Win site blockade. Second, it is possible to achieve selective inhibition of some cell types, but not others, with Win site inhibitors. Given that WDR5 itself is essential in most cancer cells [99], and that is involved in so many functions, one might naively expect Win site inhibitors to be toxic to all cells. But this is clearly not the case. These observations suggest that loss of WDR5 per se is distinct from Win site inhibition, and forecast that therapeutic windows will be achieved once drug-like inhibitors are available.

## 8. Conclusions

WDR5 is a fascinating example of a protein that sits squarely at the crossroads of development, epigenetic control, transcription, and cell division. Its multitude of interactions, with both proteins and RNAs, endows it with a broad range of molecular functions, and it appears to manage the volume of traffic at its surface by creative reuse of two main binding sites. WDR5 has emerged as a prime target for anti-cancer drug discovery efforts, which have already been shown to selectively inhibit cancer cells carrying specific genetic lesions. But what makes WDR5 such an intriguing and important protein also makes it especially challenging to study, and it is very likely that it will take many more years to fully appreciate which activities of this moonlighting master are most relevant to normal and diseased cellular states.
